# The Cognitive-Enhancing Outcomes of Caffeine and L-theanine: A Systematic Review

**DOI:** 10.7759/cureus.20828

**Published:** 2021-12-30

**Authors:** Anas Anas Sohail, Fernando Ortiz, Teresa Varghese, Stephanie P Fabara, Arshdeep S Batth, Darshan P Sandesara, Ahtesham Sabir, Mahika Khurana, Shae Datta, Urvish K Patel

**Affiliations:** 1 Medicine, Aureus University School Of Medicine, Oranjestad, ABW; 2 Neurology, Larkin Community Hospital, Miami, USA; 3 Medicine, Kasturba Medical College, Manipal University, Manipal, IND; 4 General Medicine, Universidad Catolica de Santiago de Guayaquil, Guayaquil, ECU; 5 Infectious Disease, University of Louisville, Louisville, USA; 6 Cardiology, Apex Heart Institute, Ahmedabad, IND; 7 Internal Medicine, Ayub Medical College, Abbottabad, PAK; 8 Public Health, University of California Berkeley, Berkeley, USA; 9 Neurology, NYU Langone Health, New York, USA; 10 Public Health and Neurology, Icahn School of Medicine at Mount Sinai, New York, USA

**Keywords:** caffeine, green tea, adhd, mental cognition, memory reconsolidation, l-theanine, l-theanine and caffeine, matcha

## Abstract

Attention-deficit hyperactivity disorder (ADHD) affects multiple cognitive domains, including impaired attention, hyperactivity, and increased impulsivity. According to the CDC, 9.4% of children between 2 and 17 years old have been diagnosed with ADHD. Neurotransmitters such as noradrenaline and dopamine have been suggested as crucial players in the pathophysiology of ADHD and are often targets of modern medication. Adenosine receptors types A1 and A2a in the brain are inhibited by caffeine: a stimulant known to augment attention by increasing cholinergic and dopaminergic transmission. The cognitive function of attention is also enhanced by the amino acid: L-theanine. The mechanism of action is that it behaves like a glutamate reuptake inhibitor while also acting in the hippocampus as a competitive low-affinity glutamate receptor antagonist. It’s also shown to have a neuroprotective effect by its action on the gamma aminobutyric acid (GABA)-A receptors. Our systematic review investigates the literature and clinical trials on the cognitive-enhancing effects of caffeine and L-theanine.

## Introduction and background

L-theanine is an amino acid found notably in green tea, black tea, and some mushrooms. It is known for enhancing cognitive function, particularly attention [1]. L-theanine has a few mechanisms of action. First, it is a glutamate reuptake inhibitor. Second, in the hippocampus, it is a competitive low-affinity glutamate receptor antagonist [2]. Third, it acts on the gamma aminobutyric acid (GABA)-A receptors conferring a protective effect for neurons [3]. Caffeine is a stimulant found primarily in tea, coffee, and cacao plants. Its mechanism of action includes inhibition of adenosine receptors, types A1 and A2a in the brain, which then increases cholinergic and dopaminergic transmissions, thus augmenting attention [4].

Attention-deficit hyperactivity disorder (ADHD) affects multiple cognitive domains. Patients have hindered attention, increased activity, and often act before thinking first. Combining these deficits have shed light on ADHD as a function of improper inhibitory control [5]. This systematic review will explore the impacts of L-theanine, caffeine, and particularly the combined effects of these substances on managing cognitive deficits associated with ADHD.

## Review

Methods

Protocol

Our systematic review complied with the Preferred Reporting Items for Systematic Reviews and Meta-Analysis (PRISMA) [6].

Criteria for Eligibility and Selection of Studies

Clinical trials conducted in the last 20 years were included in our review. The same criteria were used for observational studies. The findings of these studies were published in English. All other trials and studies except the ones aforementioned were excluded. Next, redundant studies in which the outcome was not appropriate to our objective were removed.

The studies we looked at compared patients to a control group which factored in outcomes such as attention enhancement, cognitive improvement, and/or ADHD.

Database and Search Strategy

PubMed was our database source. The CDC states that 9.4% of children between 2 and 17 years old have been diagnosed with ADHD [7]. Our search was conducted between 06/07/2021- 06/30/2021. We used the following search terms and their combinations: “L theanine caffeine”[Title/Abstract] OR “theanine caffeine”[Title/Abstract] OR (“Matcha”[All Fields] AND “caffeine”[Title/Abstract].

A search of MEDLINE and Scopus did not provide any additional clinical trials for this review. 

Data Extraction

Author and year of publication, methodology, and functional outcomes were collected. These studies provided the number of participants in the treatment group, the number of participants in the control group, the dose, the route of drug administration, the treatment duration, and the timing of when the drugs were given after the onset of symptoms. Modified Rankin Scale (mRS) and National Institutes of Health Stroke Scale (NIHSS) scores, Barthel Index (BI), and overall mortality are included in the baseline functional outcomes. Table 1 includes our methods summary.

**Table 1:** Methods summary

Key Terms	Database	Number of results extracted
“L theanine caffeine”[Title/Abstract]	PubMed	6
“Theanine caffeine”[Title/Abstract]	PubMed	21
“Matcha”[All Fields] AND “caffeine"[Title/Abstract]	PubMed	16

Assessment of Biases

**Figure 1:**
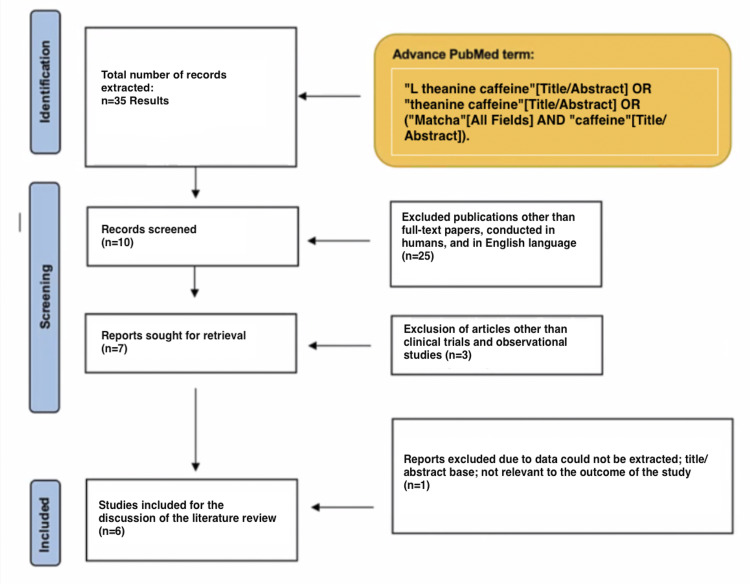
Preferred Reporting Items for Systematic Reviews and Meta-Analysis (PRISMA) flowchart

For clinical trials, we detected biases using the Cochrane collaboration’s risk of bias tool [8]. For observational studies, we used the Risk Of Bias In Non-randomized Studies--of Interventions (ROBINS-I ) tool [9]. Figure 1 uses the PRISMA flowchart to exhibit study results.

Results

These clinical trials measured one or more of the following parameters: cognition, reaction times, concentration, and/or others measure including headaches, tiredness, or alertness. Specifically, the tests used by one or more of these studies include NIH Cognition Toolbox, stop-signal reaction time (used to check control of inhibition), total cognition composite, d-prime in the Go/NoGo task, fMRI responses, mean recognition visual reaction time (RVRT), amplitudes of the mean peak-to-peak N2-P300 event-related potential (ERP), “headache” ratings, “tired” ratings, “alert” ratings, digit vigilance reaction time, correct serial seven subtractions, rapid visual information processing (RVIP) accuracy, and word recognition reaction time.

The 2020 study from Kahathuduwa et al. explored acute outcomes of L-theanine, caffeine, and their combined effects on maintained attention, control on inhibition, and general cognition in the five boys diagnosed with ADHD [10]. Improvements by the L-theanine-caffeine combination were shown on impairments related to ADHD, meaning it may be a potential therapeutic consideration. Total cognition composite was improved with L-theanine in the NIH Cognition Toolbox (p = 0.040) vs placebo. Inhibitory control was worsened by caffeine, and L-theanine, separately, as suggested by longer reaction times seen in the stop-signal intervention (p = 0.031 and p = 0.053, respectively). Whereas, improvement in cognition was seen in the total cognition composite (p = 0.041), and in the Go/NoGo task (p = 0.033). Improvements in control of inhibition (p = 0.080) was also apparent. The combination was also associated with decreased task-related reactivity in the default mode network of the brain in the region associated with mind-wandering, which meant decreased distractibility and improved concentration.

The 2018 study by Kahathuduwa et al. investigated the outcomes of 200 mg of L-theanine, 160 mg of caffeine, a fusion of the two, and distilled water in a four-way crossover study design using nine healthy adult men [11]. A visual color stimulus discrimination task was performed by the subjects, where an fMRI scan was performed for 20 minutes, 60 minutes after administering L-theanine, caffeine, and their combination. The fMRI results confirmed a decrease in mind-wandering by showing fewer responses to distractor stimuli in regions of the brain where visual attention is regulated. It was also observed that L-theanine decreases GABA levels and caffeine increases glutamate levels. This leads to the visible patterns of blood oxygenation level dependent (BOLD) responses which were seen with L-theanine alone and when combined with caffeine.

The 2017 study by Kahathuduwa et al. investigated the impact of 200 mg of L-theanine, and 160 mg of caffeine, the combination of both, a single cup of black tea, and distilled water in a five-way crossover trial with 20 healthy adult males [12]. Participants took a dose of L-theanine, which was analogous to drinking eight cups of black tea; these effects are comparable to that of caffeine. Several measurements were assessed, as mentioned in Table 2, which demonstrated an improvement in the subject’s cognitive and neurophysiological measures of selective attention after taking the combined product meaning, the additive outcomes from the L-theanine-caffeine fusion improve attention in high doses.

**Table 2:** Summary of studies involved in our systematic review ADHD: attention-deficit hyperactivity disorder; MRI: magnetic resonance imaging; NIH: National Institutes of Health; fMRI: functional magnetic resonance imaging; SVRT: simple visual reaction time; RVRT: recognition visual reaction time; ERPs: event-related potentials; VEPs: visual evoked; MEPs: motor evoked potentials; RVIP: rapid visual information processing; MMSE-J: mini-mental state examination-Japanese; UKT: Uchida-Kraepelin test

Author, year, country	Study type	Population	Study design	Intervention	Outcome
Kahathuduwa, 2020 [10]	Single-blinded, randomized placebo-controlled trial	Patients with ADHD compared to placebo. Five males between 8 and 15 years.	Participants stop caffeine and stimulants 24 hours before. Participants were evaluated using continuous performance tasks (“Go/NoGo”) and a stop-signal task in four laptops visits for 10 minutes. Functional MRI was performed during the tests	L-theanine (2.5 mg/kg) and caffeine (2 mg/kg) fusion. To evaluate NIH Cognition Toolbox	Total cognition composite was improved with l-theanine as evaluated by the NIH Cognition Toolbox (p = 0.040) vs. placebo. Both caffeine and L-theanine, individually, worsened inhibitory control shown by an augmented stop-signal reaction time; p = 0.031 and p = 0.053, respectively. Improvements in the following were noted with the combination of L-theanine and caffeine: Total cognition composite (p = 0.041), d-prime in the Go/NoGo task (p = 0.033), and inhibitory control (p = 0.080)
Kahathuduwa, 2018 [11]	Randomized control trial using a four-way crossover design	Nine healthy adult men	A visual color stimulus discrimination task was performed by subjects using four different interventions. Meanwhile, an fMRI scan was performed for 20 minutes	Using a randomized four-way crossover, L-theanine, caffeine (200 and 160 mg, respectively), a fusion of the two, and distilled water were administered accordingly. Distilled water represents a placebo	Response time to targets were quicker versus placebo (difference of 27.8 ms, p=0.018 and difference of 26.7 ms, p=0.037, respectively) were assessed with L-theanine and L-theanine-caffeine combination. Fewer fMRI signals to distractive stimuli in the brain were noted in subjects on L-theanine, where visual attention is regulated. Decreased fMRI responses to target stimuli were also assessed with the L-theanine-caffeine combination, showing a synergistic action in decreasing mind-wandering
Kahathuduwa, 2017 [12]	A placebo-controlled, five-way crossover trial	Twenty healthy males	The effects of each intervention were compared with cognitive and neurophysiological measures of attention. Cognitive measures include simple visual reaction time (SVRT) and recognition visual reaction time (RVRT). Neurophysiological measures include event-related potentials (ERPs). Treatment outcomes on visual (peripheral) and motor conduction were examined using the visual evoked potentials (VEPs) and motor evoked potentials (MEPs), respectively.	L-theanine (200 mg), caffeine (160 mg), the combination of the two, a single cup of black tea, and distilled water were administered accordingly. Distilled water represents a placebo	Significant improvement of mean RVRT was measured: L-theanine (p = 0.019), caffeine (p = 0.043), and L-theanine-caffeine fusion (p = 0.001). However, this was not the case with the following: Tea (p = 0.429) or distilled water (p = 0.822). Larger, and more significant mean peak-to-peak N2-P300 ERP were elicited by L-theanine (p = 0.001) and caffeine (p = 0.001) compared to placebo, whereas a significantly larger mean N2-P300 amplitude was recorded for the L-theanine-caffeine combination compared to placebo (p < 0.001), L-theanine (p = 0.029), or caffeine (p = 0.005)
Haskell, 2008 [13]	Randomized, placebo-controlled, double-blind, balanced crossover	Twenty-four participants completed the experiment (9 male and 15 female, mean age 21.3 years, S.E.M. 0.83, range 18–34 years). Participants abstained from caffeine and alcohol for a minimum of 12 hours before the first testing session and throughout the morning until the final testing session was completed	The acute cognitive and mood effects of the interventions were investigated in this study. Salivary caffeine levels were co-monitored	L-theanine, and caffeine (250 and 150 milligrams, respectively) were administered both individually, and combined	Augmented ratings for “headache” and fewer correct serial seven subtractions were noted in subjects taking L-theanine. Greater accuracy in rapid visual information processing (RVIP). More reports of “mental fatigue” were also assessed in subjects taking L-Theanine. These subjects also showed faster digit vigilance reaction times. Quicker simple reaction time and quicker working memory (in terms of numbers) reaction time, and better accuracy of sentence verification were features of the L-theanine-caffeine combination. Furthermore, “headache” and “tired” ratings were reduced and “alert” ratings increased. A significantly positive interaction on delayed word recognition reaction time was also noted with L-theanine-caffeine combination
Baba, 2021 [14]	Double-blind, randomized, placebo-controlled, parallel-group study	Fifty-one Japanese men and women (all healthy). Aged 50 to 69 years reported a decline in cognitive function. They also needed the ability to take nine capsules daily for 12 consecutive weeks, while having an MMSE-J score of 24 or more, and not be active smokers	Single-intake effects of the interventions, as well as continuous intake effects were compared. Under stress conditions, the effectiveness of continuous intake was assessed. Stress was instigated by the use of the Uchida-Kraepelin test (UKT). To assess cognitive function, the Cognitrax was implemented	Nine placebo, caffeine, or matcha capsules were given to participants everyday for 12 weeks before noon	During stress loading and post-stress loading, a single caffeine dose improved attentional function. Caffeine content found in matcha may have been the most likely culprit for slower reaction time in the Cognitrax. The amount of work was increased with continuous matcha intake. On the other hand, the group taking caffeine exhibited more work done for the UKT after one dose of caffeine. Work performance and attention are both improved with ingestion of matcha with caffeine during periods of psychological stress versus caffeine on its own

The 2008 study by Haskell et al. investigated the impact of 250 mg of L-theanine, and 150 mg of caffeine, individually, and in combination on the acute cognitive and mood effects in participants [13]. They found higher ratings for headache and lower correct serial seven subtractions in subjects administered L-theanine. Participants provided caffeine were noted to have quicker reaction time for the vigilance of digits, better accuracy for rapid visual information processing (RVIP), and greater reports of mental fatigue. Quicker simple reaction time, quicker working memory (in terms of numbers) reaction time, and better accuracy of sentence verification were recorded for participants taking the L-theanine and caffeine fusion. Participants reported a reduction in “headache” and “tired” ratings, while “alert” ratings were increased. Moreover, a significantly positive interaction on delayed word recognition reaction time was noted with the combination.

The 2021 study by Baba et al. evaluated the usefulness of continuous matcha intake (contains L-theanine), caffeine, their combination, and placebo under stress conditions [14]. Mild stress was induced acutely using the Uchida-Kraepelin test (UKT), while the cognitive function was evaluated by using the Cognitrax. The function of attention was improved during and post-stress loading with one caffeine dose. The caffeine content in matcha was reported to likely have caused the lower reactive time seen in the Cognitrax. However, participants continuously taking matcha showed augmented amounts of work like the caffeine group, except the caffeine group did it on a singular dose. Ingestion of the combination of matcha with caffeine improves work performance and attention under psychological stress versus with caffeine on its own.

Limitations

The 2020 study by Kahathuduwa et al. has some limitations [10]. First, it used a small sample size of only five male children with ADHD [10]. This constrains the variance of outcome measures and generalizability to all children. Second, while neuroimaging findings may have been minimally affected, the results of the Go/NoGo test, the stop-signal assignment, and the NIH Cognition Toolbox, which rely on scoring by a person involved in the study, may be influenced by bias due to unblinding [10]. Third, participants were not provided standardized food/beverages or instructions to abstain from food before each visit where they were tested [10]. Their hunger level could impact the outcomes of maintained attention, impulsive behaviors, and results seen on fMRI [15]. These limitations suggest that the preliminary evidence presented needs more power by using a larger sample size in future clinical trials.

The 2018 study by Kahathuduwa et al. was underpowered in sample size making it difficult to capture enough changes in BOLD fMRI responses from certain regions of the brain [11]. Second, only male subjects were selected in this study’s sample in order to avoid interferences from the natural changes seen in the menstrual cycle on reaction times in female subjects, thereby limiting the generalizability of this study’s findings. Third, it is well understood that caffeine affects cerebral circulation. However, cerebral circulation in human brains is not as well understood with L-theanine. Changes in blood oxygen levels in certain regions of the brain determine responses noted in BOLD fMRI. In this study, it is unclear whether the recorded observations were from true neural responses, or vascular responses which do not depend on neural activation changes. The 2017 study by Kahathuduwa et al. had technical limitations [12]. Early and late stages of processing related to attention are components of ERP, unlike EEG frequency components. Due to the technical limitations regarding baseline corrections for ERP waveforms, the amplitudes of the individual components could not be measured reliably by the study. Instead, N2-P300 peak-to-peak amplitude was measured by taking the difference between amplitudes of the N2 and the P300 peaks seen on EEG. Another limitation was the duration of reaction time tests, meaning the study was unable to evaluate the performance of the effect that treatment had on sustained attention over longer periods.

The 2008 study by Haskell et al. was limited by a lack of understanding of the mechanisms of action underlying their reported findings [13]. Whether directly or indirectly, caffeine and L-theanine have been shown to affect several neurotransmitter systems including dopamine, serotonin, glutamate, and GABA. However, at the time of their publication, the study claims, the effects of L-theanine and caffeine in combination had not been studied on the level of receptors.

The 2021 study by Baba et al. had limitations including the participants’ ages, nationality, and quality of stress [14]. The observed effects were limited to participants that were Japanese. The study selected individuals between the ages of 50 to 69 years old, who drank green tea habitually. Furthermore, the study showed a positive anti-stress effect, but only for continuous calculations of single digits to assess attention and work performance. No quantifiable biomarkers were measured, nor qualitative assessments on how stressed a participant was were performed in this study. Table 3 shows the bias risk tool analysis of each study.

**Table 3:** Shows the bias risk tool analysis of each study - = Low bias risk + = High bias risk ? = Unclear bias risk

	Random sequence generation (selection bias)	Allocation concealment (selection bias)	Blinding of participants and personnel (performance bias)	Blinding of outcome assessment	Incomplete outcome data	Selective reporting	Other biases
Kahathuduwa et al. (2020) [10]	-	-	+	-	-	-	-
Kahathuduwa et al. (2018) [11]	-	-	-	-	-	-	-
Kahathuduwa et al (2017) [12]	-	-	-	-	?	-	?
Haskell et al. (2008) [13]	-	-	-	-	-	-	?
Baba et al. (2021) [14]	-	-	-	-	-	-	?

Discussion

Overall, these clinical trials of caffeine, L-theanine, and their combination showed various functional outcomes related to attention, inhibitory control, mind-wandering, cognition, and mood [10-14].

The 2020 study from Kahathuduwa et al. showed that participants taking L-theanine demonstrated improvements in total cognition composite as seen using the NIH Cognition Toolbox (p = 0.040) compared to placebo [10]. The NIH Cognition Toolbox includes seven cognitive function tests: a flanker inhibitory control and attention test, a picture sequence memory test, a list sorting working memory test, a picture vocabulary test, an oral reading recognition test, dimensional change card sort test, and a pattern comparison processing speed test. On their own, caffeine and L-theanine showed deteriorating control on inhibition: There was an augmented reactive time for the stop-signal test for caffeine (p = 0.031) and for L-theanine (p = 0.053). However, improvements were noted with inhibitory control (p = 0.080); overall cognition composite (p = 0.041); and d-prime in the Go/NoGo assignment (p = 0.033) with subjects on the L-theanine-caffeine combination. This suggests that the combination enhances the user’s attention, cognition, and inhibitory control, perhaps via synergistic effects. Improvement in these parameters is essential for patients with ADHD, though a larger sample size is needed to increase the power of this study and prove statistical significance.

The 2018 study by Kahathuduwa et al. used fMRI on subjects to observe responses in visual color stimulus discrimination tasks [11]. L-theanine and L-theanine-caffeine fusion resulted in quicker target reactions versus placebo (difference of 27.8 ms (p = 0.018) and 26.7 ms (p = 0.037), respectively). Distractor stimuli in parts of the cerebrum where visual attention is affected showed decreased fMRI responses in participants taking L-theanine. Their results imply that the combination decreases mind-wandering, perhaps by increasing neural resources related to attention toward target stimulus, and lowering neural resources toward distractions. This would explain why the combination helps increase the user’s attention on a given task. However, the study also needs a larger sample size, ideally including both men and women. Furthermore, caffeine’s effect on cerebral circulation needs to be taken into account before making generalizations about the combination's perceived favorable effects.

The 2017 study by Kahathuduwa et al. showed mean recognition visual reaction time (RVRT) was significantly improved by L-theanine (p = 0.019), caffeine (p = 0.043), and L-theanine-caffeine combination (p = 0.001), but not by tea (p = 0.429) or placebo (p = 0.822) [12]. The ERP is a time-locked measure of the electrical activity of the cerebral surface representing a distinct phase of cortical processing. Two components of the ERP which bear special importance to stimulus evaluation, selective attention, and conscious discrimination in humans are the P300 positivity and N200 negativity. Amplitudes of the mean peak-to-peak N2-P300 ERP were significantly larger when elicited with L-theanine (p = 0.001) and caffeine (p = 0.001) versus placebo; whereas a significantly larger mean N2-P300 amplitude was measured with L-theanine-caffeine combination compared to placebo (p < 0.001), L-theanine (p = 0.029), or caffeine (p = 0.005). This means visual and motor conduction improved significantly with the combination. However, this approach was limited by its duration of only 10 target trials in each reaction time test. Therefore, the study could not evaluate the effect that treatment had on the performance of sustained attention over a longer period. Further investigation is still needed on the length of sustained attention when taking the L-theanine-caffeine combination.

The 2008 study by Haskell et al. showed higher ratings for headache and lower correct serial seven subtractions in subjects taking L-theanine alone [13]. Whereas improved RVIP accuracy, faster digit vigilance reaction time, and attenuated increases in mental fatigue (self-reported) were noted in subjects taking caffeine alone. Quicker simple reaction time and quicker working memory (in terms of numbers) reaction time, and better accuracy of sentence verification were features of the L-theanine-caffeine combination. Ratings of 'headache' and tiredness were reduced, while ratings of alertness were increased. A significantly positive interaction on the reaction time of delayed word recognition was also measured in participants taking the fusion of L-theanine and caffeine. Although promising, research is still needed to further understand the neurochemical effects of L-theanine and caffeine in combination at the receptor level to better explain these findings.

The 2021 study by Baba et al. showed improved function in attention both during and post-stress loading with a single dose of caffeine [14]. Mild stress was induced using the Uchida-Kraepelin test (UKT), while the function of cognition was evaluated using the Cognitrax. The Cognitrax is an assessment procedure that uses reliable computerized neuropsychological tests to evaluate the neurocognitive status of patients covering a range of mental processes from simple motor performance, attention, memory, to processing speed. The Cognitrax showed reduced reaction times when observing participants that took a single dose of matcha. Matcha’s caffeine content may have been the underlying cause for the observation. Participants that continuously took L-theanine (by ingestion of matcha) exhibited more amounts of work completed while UKT was administered, similar to those on a single dose of caffeine. The researchers concluded that there was improved attention and work performance for participants experiencing induced psychological stress when taking matcha in combination with caffeine versus with caffeine alone. However, the results may be skewed as many participants already had a habit of drinking green tea. Furthermore, since no quantifiable biomarkers were measured, nor qualitative assessments to assess a participant’s level of perceived stress, further investigation on the combination’s effect on stress, performance, attention, memory, and overall cognition is advised.

## Conclusions

Caffeine and L-theanine are natural compounds found primarily in tea and coffee, respectively. The combination has shown improvement in short-term sustained attention and overall cognition. Reversed task-related mind-wandering and improved inhibitory control were also seen among boys with ADHD, while improvements in mild acute stress and increased amount of work were noted in the population of men and women aged 50-69 in Japan. After reviewing the studies, we found the combination shows favorable clinical significance in the domains of attention, memory, cognition, and hyperactivity. Overall, we conclude that the combination of L-theanine and caffeine is likely a safe and effective cognitive enhancer. Further research is still needed to explain the aforementioned limitations.
